# Diagnostic and Therapeutic Challenges in Rare and Non-Tubal Ectopic Pregnancies: A Narrative Review

**DOI:** 10.3390/diagnostics16050793

**Published:** 2026-03-07

**Authors:** Stefan Ivanovic, Milica Ivanovic, Dragana Maglic, Milica Mandic, Lidija Tulic, Katarina Ivanovic, Milos Milincic, Nikola Jovic, Rastko Maglic

**Affiliations:** 1 Obstetrics and Gynecology Clinic “Narodni Front”, 11000 Belgrade, Serbia; 2 Faculty of Medicine, University of Belgrade, 11000 Belgrade, Serbia; 3 Clinic for Gynecology and Obstetrics, University Clinical Center of Serbia, 11000 Belgrade, Serbia; 4 Department of Gynecology and Obstetrics, Faculty of Medical Sciences, University of Kragujevac, 34000 Kragujevac, Serbia; 5 University Clinical Center Kragujevac, ZmajJovina 30, 34000 Kragujevac, Serbia

**Keywords:** ectopic pregnancy, non-tubal ectopic pregnancy, pregnancy of unknown location, transvaginal ultrasound, diagnostic pitfalls

## Abstract

In relation to the most commonly described ampullary ectopic pregnancies in contemporary gynecological practice, rare localizations of ectopic pregnancies represent a diagnostic and therapeutic challenge whose clinical significance far exceeds their frequency. In contrast to tubal ectopic pregnancy, these implantation localizations are characterized by specific anatomical relationships and early trophoblastic invasion into highly vascularized tissues, which is why classical diagnostic algorithms and therapeutic patterns are often not applicable in clinical practice. Clinical uncertainty is further increased by the fact that a large proportion of these pregnancies in early gestation cannot be precisely mapped and initially present as pregnancies of unknown location. This narrative review integrates contemporary evidence and guidelines of relevant professional societies with the aim of highlighting patterns of diagnostic errors, systemic weaknesses of existing approaches, and key points for safe clinical decision-making. Special emphasis is placed on the role of disciplined transvaginal ultrasound evaluation, terminological precision, and timely recognition of high-risk and nonspecific implantations. Analysis of the available literature indicates that therapeutic decisions must be individualized and guided by the implantation site and assessment of hemorrhagic risk, rather than gestational age or absolute β-hCG values. Understanding these principles represents the basis for reducing serious complications and for the development of future diagnostic and therapeutic algorithms, thereby improving treatment outcomes.

## 1. Introduction

Ectopic pregnancy (EP) is defined as implantation of gestational tissue outside the endometrium, at an abnormal site, regardless of whether the implantation location is situated outside or within the uterus [1]. Adequate implantation represents trophoblastic invasion that does not extend beyond the endometrial–myometrial junction, and contemporary terminological frameworks are increasingly focused on precise localization and implantation pattern rather than on a binary distinction of “intrauterine versus extrauterine” implantation [1,2]. In this context, the recommendations of the European Society of Human Reproduction and Embryology (ESHRE) emphasize the need for standardized ultrasound (US) terminology in early gestation in order to reduce diagnostic errors and enable safe clinical decision-making [1]. Of particular clinical importance are abnormal implantations that may be located within the uterus, such as cesarean scar pregnancy (CSP), cervical pregnancy, or intramural pregnancy, since misinterpretation of their “intrauterine” localization may lead to delayed diagnosis and the development of serious complications [1,3,4]. The estimated incidence is approximately 2% of all pregnancies. EP remains one of the leading causes of maternal morbidity in the first trimester of pregnancy [4,5,6]. The pathogenesis of EP is multifactorial and most commonly associated with impaired transport of the fertilized ovum and/or local conditions that favor implantation outside the physiological endometrial environment. In clinical practice, the risk is increased in patients with a history of EP, pelvic inflammatory disease, tubal damage (surgical or inflammatory), infertility, and the use of assisted reproductive technologies (ART), while in a proportion of patients the predisposing factor remains unrecognized [5,6,7]. The clinical presentation of rare EP localizations is often nonspecific. Lower abdominal or pelvic pain and vaginal bleeding may be minimal or absent, whereas hemodynamic instability may develop abruptly, particularly in localizations with high hemorrhagic potential [5,6,8]. Although the majority of EPs are of tubal origin, the clinical focus of this review is directed toward non-standard, non-ampullary, and rare localizations, which are disproportionately important due to the higher risk of incorrect or delayed diagnosis, as well as specific diagnostic and therapeutic limitations [3,4,7]. The aim of this review is to integrate contemporary evidence and the positions of relevant professional guidelines regarding rare EP localizations, with particular emphasis on diagnostic challenges, clinical pitfalls, and therapeutic strategies that differ from the approach to typical ampullary tubal EP, with the goal of improving gynecological practice [1,2,3,4,5,6,7].

## 2. Methods

A literature search was conducted in PubMed/MEDLINE, Scopus, and Web of Science using a structured but non-systematic approach adapted to the pronounced heterogeneity of available data on rare and non-tubal ectopic pregnancies. Search terms were combined into thematic groups to encompass different implantation sites and diagnostic concepts and included the following keywords: “ectopic pregnancy”, “non-tubal ectopic pregnancy”, “cesarean scar pregnancy”, “cervical ectopic pregnancy”, “interstitial pregnancy”, “cornual pregnancy”, “ovarian pregnancy”, “abdominal pregnancy”, “heterotopic pregnancy”, “pregnancy of unknown location”, and “transvaginal ultrasound”.

The search included full-text articles published in English, involving human subjects and indexed from January 2015 onward. Older publications were selectively included only when relevant to terminological definitions, classification, or fundamental diagnostic and conceptual frameworks.

### 2.1. Inclusion Criteria and Study Selection

Studies were included if they met one or more of the following criteria:

1. Described diagnostic criteria or imaging features relevant to rare and non-tubal ectopic pregnancy localizations;
2. Analyzed therapeutic approaches or clinical outcomes specific to non-tubal ectopic pregnancies;
3. Contained recommendations based on professional society guidelines or expert consensus;
4. Addressed methodological limitations, epidemiological trends, or short- and long-term reproductive outcomes.

Studies focusing exclusively on standard tubal ectopic pregnancy (most commonly ampullary localization) were excluded, except when used for conceptual comparison. Publications not available in English were not included in the analysis.

**Figure 1:**
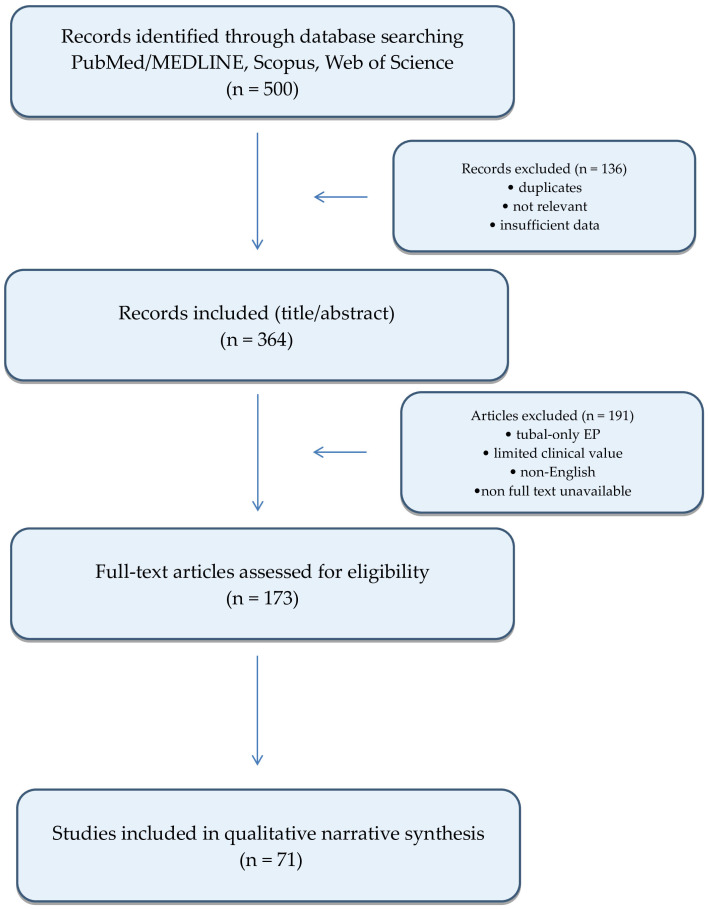
Prisma-like model.

The initial database search yielded approximately 500 publications. Following screening and eligibility assessment, studies lacking direct diagnostic or therapeutic relevance, as well as isolated case reports without additional clinical value, were excluded. Individual case reports and small case series were selectively included, primarily in the context of exceptionally rare localizations or when they provided unique diagnostic or therapeutic insights. A total of 71 publications were ultimately included in the final narrative synthesis. The study selection process is summarized in a PRISMA-like flow diagram (Figure 1).

### 2.2. Data Extraction and Synthesis

Relevant data were extracted qualitatively and included the following domains: implantation localization and classification, diagnostic approaches and ultrasound criteria, common diagnostic errors and causes of delayed diagnosis, therapeutic modalities and their limitations, and short- and long-term clinical outcomes.

Data synthesis was performed narratively and thematically, without quantitative analysis. Findings were organized into structured sections addressing epidemiological limitations, diagnostic patterns, therapeutic challenges, fertility implications, and gaps in the existing evidence base. Particular emphasis was placed on retrospective studies, heterogeneous case series, and expert opinions, which represent the predominant sources of evidence in this field.

Quantitative meta-analysis was not performed, as it could have resulted in inaccurate or potentially misleading conclusions given the marked heterogeneity of study designs, diagnostic criteria, therapeutic strategies, and reported outcomes across rare ectopic pregnancy localizations. The principal limitation of this research is its narrative design rather than a systematic review; however, a systematic methodology with quantitative synthesis was not feasible due to the lack of sufficiently homogeneous cohort studies and comparable outcome data. Under such circumstances, quantitative aggregation could create a false impression of precision without corresponding clinical validity. Therefore, a narrative review was considered the most appropriate methodological framework for integrating available evidence and identifying clinically relevant diagnostic and therapeutic patterns based on implantation site.

Review and interpretation of relevant sources were performed independently by two authors. Potential ambiguities, differences in interpretation, and possible overlaps in data or publications by the same authors were critically assessed and resolved through joint discussion and consensus.

## 3. Results

The primary objective of first-trimester US examination is confirmation of a viable intrauterine pregnancy and precise localization of implantation. TVUS represents the cornerstone of contemporary early pregnancy diagnostics, while current guidelines of leading gynecological societies further emphasize standardized reporting and the use of clear terminology in situations in which immediate confirmation of an intrauterine pregnancy or EP is not possible [1,2,3,5]. To address the well-documented heterogeneity in terminology, standardized definitions were applied throughout this manuscript in accordance with ESHRE and ISUOG recommendations. The term interstitial pregnancy refers exclusively to implantation within the intramural portion of the fallopian tube at the uterotubal junction, whereas cornual pregnancy is reserved for pregnancies associated with uterine anomalies (rudimentary uterine horn). Angular pregnancy is considered an intrauterine implantation and is not classified as an ectopic pregnancy (Table 1) [1,3,5].

The literature identifies the following entities as the most clinically significant non-standard EP localizations:

(i) Cesarean scar pregnancy (CSP);
(ii) Cervical ectopic pregnancy;
(iii) Interstitial pregnancy;
(iv) Cornual pregnancy in the context of uterine anomalies;
(v) Ovarian pregnancy;
(vi) Abdominal pregnancy, including broad-ligament and other ligamentous forms [1,5,7,8].

Rare EPs encompass a heterogeneous group of localizations and are summarized in Table 2.

Although they collectively account for a small proportion of all EPs, these entities have been described in the literature as being associated with an increased risk of delayed diagnosis, massive hemorrhage, emergency surgical interventions, and permanent impairment of fertility and overall health, rendering them disproportionately important in everyday clinical practice [2,3,9].

### 3.1. Heterogeneity of the Term “Rare Localizations” in Contemporary Literature

Analysis of contemporary literature demonstrates that the term “rare localizations of ectopic pregnancy” is not uniformly defined and is used in a heterogeneous and context-dependent manner [1,2,3,4]. In some sources, this term refers exclusively to extremely rare entities, such as abdominal or broad-ligament pregnancy, whereas in other publications it encompasses a broader spectrum of non-standard implantations that deviate from the classical tubal model, including CSP, cervical ectopic pregnancy, and interstitial pregnancy [2,5,6]. An increasing number of authors emphasize that the concept of “rarity” in this context does not relate solely to low incidence, but rather primarily to the absence of standardized diagnostic and therapeutic algorithms, limited availability of reliable evidence, and increased clinical risk compared with typical tubal EP [3,7]. This perspective explains why certain entities, such as CSP, continue to be classified as “rare” despite a documented increase in their incidence, given that they still account for a small proportion of the overall number of EPs and of all pregnancies [8,9,10]. Within this group are included localizations with markedly different epidemiological characteristics, clinical courses, and diagnostic–therapeutic challenges, which further contributes to inconsistent use of the term in the literature [4,6,11]. This heterogeneity is also clearly reflected in the structure of the available evidence, which is largely based on individual case reports, small series, and a limited number of reviews, thereby significantly hindering direct comparison of results and the formulation of universal conclusions [7,12,13].

### 3.2. Pregnancy of Unknown Location (PUL) as a Conceptual Framework for the Detection of Rare Ectopic Pregnancies

According to the literature, PUL is defined as a condition in which, after the initial TVUS examination, neither intrauterine pregnancy nor EP can be confirmed in the presence of a positive serum β-hCG level [7,14]. Guidelines consistently emphasize that PUL does not represent a diagnosis, but rather a temporary US-based classification of early pregnancy outcome, which requires structured and dynamic follow-up until definitive diagnostic resolution is achieved [14,15,16]. The PUL concept has been standardized and elaborated in multiple guidelines and consensus documents and involves a combination of serial serum β-hCG monitoring and repeated TVUS examinations, with the aim of classifying the pregnancy into one of three outcome categories: 1. intrauterine pregnancy, 2. failed PUL and 3. EP [14,15,16]. The literature emphasizes that this process must be conducted with strict assessment of clinical stability and exclusively in hemodynamically stable patients, since both ectopic and normally developing pregnancies may occur even at relatively low or slowly rising β-hCG levels [15,16,17,18]. In rare ectopic localizations, early US signs of EP are frequently absent, as implantation occurs outside anatomical regions that are routinely within the focus of the initial examination [8,17,19]. Consequently, a significant proportion of these pregnancies remain classified as PUL in the early stage, which explains why PUL algorithms have particular clinical value in the early identification of non-standard and rare EPs [7,16,20]. The literature indicates that a substantial proportion of rare EPs clinically manifest precisely within the PUL category in the early phase, particularly CSP, interstitial pregnancy, and heterotopic pregnancy [8,15,17]. In addition to standard serial β-hCG monitoring, predictive models based on dynamic changes in hCG values, such as the M4 and M6 models, have been described in the literature with the aim of early risk assessment for EP within the PUL population [21,22,23]. Although these models have been validated and may be useful for initial risk stratification, the literature consistently indicates that they cannot replace clinical assessment, serial TVUS examinations, and serial monitoring of β-hCG dynamics, particularly in situations where rare ectopic localizations are suspected [22,23,24]. Taken together, these findings confirm that PUL is regarded in contemporary literature as an initial phase of the diagnostic process that enables safe patient follow-up and timely recognition of EP before the development of serious complications [7,15,17].

### 3.3. Diagnostic Patterns Identified in the Literature

#### 3.3.1. Limitations of Available Epidemiological Data on Rare Ectopic Pregnancies

Epidemiological data on rare EP localizations in contemporary literature are characterized by numerous methodological limitations, as consistently emphasized in review articles and professional society guidelines [4,5,6,7]. Unlike tubal EP, for which reliable data are available from national registries and large cohort studies, most information on rare localizations derives from retrospective studies, systematic reviews, and individual case reports or small case series [5,6,7]. One of the key issues identified in the literature is pronounced underreporting of rare EPs, particularly those that resolve in early gestation or are initially classified as PUL [7,14,25]. Several authors report that a proportion of these pregnancies resolve spontaneously or are diagnosed only intraoperatively, leading to systematic underestimation of their true incidence in the general population [6,9,26]. An additional limitation is inconsistent terminology and classification, particularly with regard to implantations in the interstitial portion of the fallopian tube and cervico-isthmic localizations [9,27,28]. Differences in definitions across studies, as well as documented misinterpretation of implantation sites in certain publications (e.g., conflation of cornual and interstitial pregnancy), preclude direct comparison of incidence and outcomes, resulting in substantial variability in the reported rates of specific localizations [10,29,30,31,32]. Although this issue is more pronounced in older literature, it remains present in contemporary studies that do not apply standardized US criteria and classifications recommended by leading gynecological and ultrasonography societies [28,30,33]. The most common diagnostic challenges and systematic errors in the identification of rare EPs, together with their potential clinical consequences, are summarized in Table 3. The literature also points to a marked publication bias, as rare and clinically dramatic presentations are more frequently published as individual case reports or small case series, whereas less complicated or subclinical forms often remain unreported. Consequently, the available data tend to overestimate clinical severity and the risk of complications, further complicating accurate epidemiological assessment [5,13,34]. Another important limitation relates to differences between tertiary referral centers and secondary care institutions, as well as discrepancies relative to the general population. Several studies emphasize that data from tertiary centers cannot be directly generalized, as these centers are disproportionately burdened with complex and complicated cases compared with secondary centers; this selection bias significantly affects reported incidence rates and clinical outcomes [4,6,7].

#### 3.3.2. Changes in the Epidemiological Profile of Rare Ectopic Pregnancies in Contemporary Clinical Practice

Contemporary literature indicates that the epidemiological profile of EP has changed over recent decades, with these changes being particularly pronounced in rare and non-standard localizations [5,8,35,36,37]. The most commonly cited dominant factors include an increase in cesarean section rates and wider use of ART, with an additional contribution from the growing frequency of uterine interventions and invasive diagnostic and therapeutic procedures [7,38,39]. The increase in the incidence of CSP reported in literature is almost universally associated with the global rise in cesarean section rates [40,41,42]. Multiple sources emphasize that CSP represents a relatively new clinical entity in historical terms, becoming clearly recognizable only with the development of high-resolution TVUS and the increasing number of women with a history of cesarean delivery [7,10,40]. Although CSP continues to be classified as a rare localization, available data indicate a rising frequency, particularly in tertiary centers, with direct implications for everyday clinical practice [7,10,40,43]. A similar epidemiological pattern has been observed for heterotopic pregnancy, the incidence of which increases significantly in populations of women undergoing ART [14,44,45]. In contrast, in spontaneous cycles, heterotopic pregnancy is described as exceedingly rare, whereas some studies report a several-fold higher incidence in ART populations. This increase is attributed to ovulation induction, transfer of multiple embryos, and altered tubal function [7,46,47,48]. This trend has led to increased clinical vigilance and the need for systematic early US evaluation of the adnexa, even after confirmation of an intrauterine pregnancy in ART cycles [12,49,50,51,52]. The literature also describes an association between prior uterine interventions and the occurrence of cervical and cervico-isthmic EPs [35,53,54,55]. Although the available data are heterogeneous, several review articles suggest that previous curettage, hysteroscopic procedures, and other intrauterine interventions may contribute to alterations in endometrial and cervical architecture, thereby creating conditions favorable for non-standard implantation [50,51,52,53,54]. However, contemporary sources emphasize that this association is largely based on retrospective analyses and does not allow reliable prediction of individual risk, as these rare pregnancies also occur in patients without clearly defined risk factors [50,53]. Interstitial EP is increasingly diagnosed at earlier gestational ages in contemporary literature, a trend attributed to advances in US techniques and greater clinician awareness of this entity [28,29,31,34]. Despite earlier recognition, available data indicate that interstitial pregnancy continues to carry a significantly higher risk of rupture and severe hemorrhage compared with tubal EP [21,28,29,31]. Finally, modern literature emphasizes that observed changes in the epidemiological profile of rare EP should not be interpreted exclusively as a true increase in incidence, but also as a consequence of improved diagnostic capabilities, wider availability of early US, and more precise diagnostic criteria [7,21,22,29]. At the same time, the lack of uniform registries and standardized reporting precludes accurate quantification of these trends for individual rare localizations [7,40,42]. Because of these methodological limitations, most sources agree that available epidemiological estimates should be interpreted with caution and within the context of the limitations from which they arise [5,6,7].

### 3.4. Diagnostic Characteristics of Rare Ectopic Pregnancies

#### 3.4.1. General Diagnostic Principles and Limitations of the Standard Approach

Modern studies and clinical guidelines agree that the fundamental diagnostic approach to EP is based on a combination of clinical assessment, TVUS, and serial monitoring of serum β-hCG levels [1,2,3,4]. However, the literature consistently emphasizes that diagnostic criteria developed for tubal, most commonly ampullary, EP cannot be reliably applied to rare and non-standard localizations [2,5,7]. In rare EP, US findings in early gestation are often inconclusive, as classic signs of EP—such as an adnexal mass, free intraperitoneal fluid, or “tubal ring” sign—may be absent or atypical [3,6,21]. As a result, the initial US examination in a substantial proportion of cases concludes with classification of the pregnancy as PUL, which in this context does not represent a diagnostic failure but rather a safe and rational step that enables structured follow-up and timely identification of the implantation site [7,9,14]. The literature further indicates that β-hCG values in rare EP often overlap with those observed in normal intrauterine pregnancies or in early non-progressive pregnancies; therefore, the diagnostic value of isolated laboratory findings is substantially reduced, particularly when they are not interpreted in correlation with clinical and US findings [14,15,18].

#### 3.4.2. Role of Transvaginal Ultrasound in the Diagnosis of Rare Localizations

TVUS remains the primary diagnostic modality in the evaluation of early pregnancies. However, contemporary literature emphasizes that when rare ectopic localizations are suspected, TVUS examination must be performed in a targeted and systematic manner, particularly in patients with identified risk factors [7,11,21]. In uterine EP, particular attention is directed toward the relationship of the gestational sac to the endometrial cavity, myometrium, and cervical canal. In addition, routine use of color Doppler techniques is recommended to assess peritrophoblastic vascularization and the potential risk of hemorrhage [21,22,25,46]. Several authors note that reliable localization of implantation in these entities is often achieved only during serial TVUS examinations, especially in very early gestations [10,14,21]. Accordingly, when rare EP is suspected, an expanded US protocol is frequently required, extending beyond standard adnexal assessment to include detailed evaluation of the lower uterine segment, cervix, and the region of a previous cesarean section [8,11,46].

#### 3.4.3. Diagnostic Characteristics of Cesarean Scar Pregnancy (CSP)

In cases of CSP, modern literature indicates that the diagnosis is primarily established during the first trimester of gestation based on well-defined TVUS criteria [40,46,47]. The most commonly reported sonographic features include implantation of the gestational sac at the site of a previous cesarean section, absence or minimal thickness of the myometrial layer between the gestational sac and the serosa, and lack of communication between the gestational sac and the endometrial cavity [40,46,47,49]. Color Doppler assessment is widely recognized as an important adjunctive tool, as it enables evaluation of peritrophoblastic vascularization and facilitates differentiation of CSP from a low-implanted intrauterine pregnancy or an ongoing miscarriage [21,22,40,46]. Despite the availability of standardized diagnostic criteria, several sources emphasize that these criteria are not applied uniformly across published studies, resulting in heterogeneity of reported US findings and variability in diagnostic accuracy [40,47,48]. From a conceptual standpoint, implantation in a cesarean scar should be understood as a form of pathological implantation within scar-altered myometrium rather than solely as a direct consequence of prior cesarean delivery. Contemporary reports indicate that similar implantation patterns may also occur in areas of myometrial disruption following other uterine surgical procedures, including myomectomy and reconstructive uterine surgery [40,51,52]. This broader perspective underscores the importance of precise anatomical localization and careful assessment of the relationship between the gestational sac, the myometrium, and surrounding vascular structures during the diagnostic process [21,22,40,51,52].

#### 3.4.4. Diagnostic Characteristics of Cervical and Cervico-Isthmic Pregnancy

Cervical EP is described in the literature as diagnostically particularly challenging, primarily due to its clinical and US similarity to an ongoing miscarriage [22,23,24]. The classic US finding includes the presence of a gestational sac within the cervical canal, with absence of an intrauterine gestation within the uterine corpus [10,22,50]. One frequently cited diagnostic feature is the absence of the “sliding sign,” which may assist in differentiating cervical pregnancy from an ongoing miscarriage [23,25]. Color Doppler techniques are also reported to be useful for assessment of vascularization of the cervical stroma and trophoblast, particularly in evaluating the risk of bleeding [26,54,55]. Cervico-isthmic pregnancy is increasingly recognized in contemporary studies as a distinct entity, with implantation in the transitional zone between the cervix and the lower uterine segment [27,29,55]. The literature emphasizes that the boundary between cervical and cervico-isthmic pregnancy is not always clearly defined, which may lead to variability in diagnostic classification and reported clinical outcomes [29,31,32].

#### 3.4.5. Interstitial Pregnancy and Cornual Pregnancy: Diagnostic Features and Terminological Considerations

Interstitial pregnancy is described in the literature as a particularly high-risk localization due to frequent delayed diagnosis and the possibility of rupture at more advanced gestational ages, often accompanied by massive hemorrhage [21,30,31,32]. Diagnostic criteria are based on identification of the gestational sac within the intramural segment of the fallopian tube, lateral to the endometrial cavity, with the presence of a thin myometrial layer surrounding the gestational sac [11,31,56]. Several sources describe the “interstitial line sign” as a potentially useful TVUS finding and literature indicates that its sensitivity and specificity vary depending on the experience of the ultrasonographer and the gestational age at which the examination is performed [31,33,34]. An additional diagnostic challenge is terminological overlap with angular (intrauterine, not ectopic) and cornual pregnancies, which has been extensively addressed in contemporary review articles and represents a frequent source of diagnostic and therapeutic uncertainty [29,30,32].

#### 3.4.6. Diagnostic Characteristics of Ovarian Ectopic Pregnancy

Ovarian EP is described in the literature as a diagnostically particularly challenging localization, primarily due to its pronounced ultrasonographic similarity to corpus luteum cysts and other hemorrhagic ovarian lesions [24,35,36,37,57]. Typical US findings include a solid–cystic adnexal mass in the projection of the ovary, frequently associated with marked peripheral vascularization, commonly referred to as the “ring of fire” sign, which substantially complicates differentiation from benign ovarian structures [24,36,38,57]. Importantly, these sonographic features are not specific and may also be observed in physiological or benign ovarian conditions encountered in early pregnancy. Consequently, clinical suspicion should be heightened when an adnexal lesion demonstrates atypical morphology, disproportionate vascularization, or sonographic characteristics that are discordant with the expected appearance and temporal evolution of a physiological corpus luteum. Multiple sources report that, due to these diagnostic ambiguities, definitive diagnosis of ovarian EP is established in a substantial proportion of cases only intraoperatively or on the basis of histopathological examination, which further complicates epidemiological assessment and contributes to underestimation of this entity in routine clinical practice [24,35,37,57]. Historically, the diagnosis of primary ovarian EP was based on the classical Spiegelberg criteria, which include the presence of an intact ipsilateral fallopian tube, localization of the gestational sac within the ovary, and histological confirmation of ovarian tissue attached to the pregnancy specimen. Although these criteria continue to be referenced in contemporary literature, their practical value in modern clinical settings is considered limited, as most cases are now initially suspected on the basis of US findings and confirmed intraoperatively or histopathologically [56,57,58].

#### 3.4.7. Diagnostic Characteristics of Abdominal and Ligamentous Pregnancy

Abdominal and ligamentous EPs are described in the literature as exceedingly rare localizations that are frequently unrecognized in the early stages of gestation [39,40,41,42,57]. Their atypical implantation outside anatomical regions that are routinely evaluated during standard TVUS examination represents a significant diagnostic challenge and requires a high level of clinical suspicion [40,57,58]. US findings in these cases are most often nonspecific and may include absence of an intrauterine gestation with the presence of a gestational structure in an atypical intra-abdominal location, necessitating careful and targeted interpretation of imaging findings [41,43,59]. Several review articles report that the diagnosis of abdominal pregnancy is not infrequently established intraoperatively, particularly in advanced cases or in emergency clinical situations accompanied by hemoperitoneum and hemodynamic instability [41,42,59,60].

#### 3.4.8. Diagnostic Challenges of Heterotopic Pregnancy

In heterotopic pregnancy, literature emphasizes that the presence of a confirmed intrauterine pregnancy may lead to delayed diagnosis of the concomitant ectopic component [43,44,45]. This diagnostic challenge most commonly arises from reduced clinical suspicion following documentation of intrauterine gestation, which may result in insufficiently detailed or incomplete assessment of the adnexa [21,45]. Review articles and clinical recommendations indicate that systematic evaluation of the adnexa is necessary even in situations where an intrauterine pregnancy has been clearly confirmed, particularly in patients with a history of ART use, in whom the risk of heterotopic pregnancy is significantly increased [21,43,44,45].

### 3.5. Therapeutic Patterns in Rare Ectopic Pregnancies

#### 3.5.1. General Therapeutic Principles and Limitations of the Standard Tubal Approach

The current literature indicates that therapeutic management of EP has traditionally been based on algorithms developed for tubal localization, including expectant management, medical treatment with methotrexate (MTX), and surgical intervention [8,9,10,11]. It should be noted that the use of MTX in the treatment of EP represents off-label use in many countries. Therefore, patients must be appropriately counseled and informed about the off-label nature of this therapy, including potential risks, benefits, and alternative treatment options prior to its administration [9,10]. However, review articles and clinical guidelines emphasize that these algorithms cannot be directly applied to rare and non-standard localizations, given differences in implantation anatomy, specific hemorrhagic risk, and limited data on therapeutic outcomes [5,7,40,42]. In rare EP, therapeutic decision-making is described in the literature as being primarily determined by the patient’s hemodynamic status, precise localization of implantation, and availability of surgical and interventional modalities, whereas gestational age and absolute serum β-hCG values have a substantially more limited predictive value compared with standard tubal EP [7,15,16,42]. Therapeutic patterns in rare EPs vary considerably depending on implantation site, as summarized in Table 4.

#### 3.5.2. Therapeutic Patterns in Cesarean Scar Pregnancy

In CSP, available literature and consensus documents consistently indicate that expectant management carries a high risk of serious complications and is therefore not considered an acceptable therapeutic option in most cases [40,42,47]. Systemic MTX used as monotherapy has likewise demonstrated limited effectiveness and is frequently associated with a prolonged disease course and the need for additional therapeutic interventions [40,42,48]. Accordingly, the most commonly reported therapeutic strategies involve localization-guided, procedure-based approaches. These include surgical removal of gestational tissue via transvaginal (often hysteroscopically guided) or laparoscopically guided techniques, while laparotomy is generally reserved for selected cases, such as those complicated by hemodynamic instability or suspected massive hemorrhage. Ultrasound-guided aspiration and combined strategies incorporating local (intragestational) administration of MTX as an adjuvant measure have also been described in carefully selected patients [40,42,46,47,52]. Several authors emphasize that isolated curettage performed without prior assessment of local vascularization and the relationship between the implantation site and the myometrium carries a substantial risk of uncontrolled hemorrhage and should therefore be avoided as a standalone therapeutic strategy [40,48,52,54]. Beyond removal of gestational tissue, assessment—and, when appropriate, correction—of the underlying scar defect may be considered in selected cases, particularly in centers with relevant expertise, with the aim of reducing the risk of recurrent pathological implantation. Taken together, these considerations support a therapeutic approach that is individualized and guided primarily by precise localization of implantation and anatomical relationships, rather than reliance on biochemical parameters or extrapolation of algorithms developed for tubal ectopic pregnancy [40,47].

#### 3.5.3. Therapeutic Patterns in Cervical and Cervico-Isthmic Pregnancy

The literature on cervical EP almost uniformly emphasizes that therapeutic management must be primarily directed toward hemorrhage control, given the pronounced vascularization of cervical anatomy [53,54,55,56]. Expectant management is rarely described, whereas conservative, fertility-preserving strategies are applied in the majority of reported cases [54,55]. The most commonly reported therapeutic modalities include combinations of ultrasound-guided aspiration, local and/or systemic MTX administration, and adjuvant hemostatic procedures such as uterine artery embolization [50,52,53]. In selected reports, hysteroscopically guided removal of gestational tissue is also described, most often as part of a combined therapeutic approach in carefully selected cases [50,52]. The literature indicates that cervico-isthmic pregnancy is most commonly managed in clinical practice according to therapeutic principles similar to those applied to other uterine EPs, albeit without a single standardized therapeutic approach [52,55]. This heterogeneity of applied strategies reflects limited available evidence and inconsistent classification of this entity in contemporary literature [29,31,50].

#### 3.5.4. Therapeutic Patterns in Interstitial Pregnancy and Cornual Pregnancy

In interstitial EP, articles describe a wide spectrum of therapeutic approaches, including medical treatment, various surgical techniques, and combined strategies [30,31,34]. A clear trend toward minimally invasive surgery, particularly laparoscopic procedures, is noted in hemodynamically stable patients, with the aim of reducing morbidity and preserving reproductive potential [30,31,34]. MTX is reported as a therapeutic option in carefully selected cases in early gestation, with multiple authors emphasizing that the success of conservative treatment largely depends on precise diagnostic classification of implantation and that the risk of rupture remains present even during therapy [30,31,48]. Terminological overlap with angular and cornual pregnancies further complicates interpretation of therapeutic outcomes in published studies, as these entities are inconsistently defined and frequently grouped together in the available literature [29,30,32].

#### 3.5.5. Therapeutic Patterns in Ovarian Ectopic Pregnancy

Ovarian EP is most commonly described as an entity managed surgically, with definitive diagnosis established intraoperatively in a substantial proportion of cases [24,35,36,37,57]. Contemporary series indicate that conservative surgical procedures aimed at preservation of ovarian tissue represent the most frequently reported therapeutic approach, particularly in patients with a strong desire to preserve fertility [35,37,45]. In contrast, medical treatment with MTX has been described only in isolated case reports; however, the available literature consistently highlights a limited evidence base and heterogeneous therapeutic outcomes, precluding reliable conclusions regarding its effectiveness in the management of ovarian EP [35,37,48,57]. Consequently, conservative medical management has a very limited role, while surgical treatment remains the predominant therapeutic strategy in most clinical scenarios. Available data further suggest that the extent and type of surgical intervention are most often determined intraoperatively, based on the degree of ovarian tissue involvement and the patient’s hemodynamic status, rather than on predefined algorithms or isolated biochemical parameters. This therapeutic pattern underscores the importance of individualized, anatomy-driven decision-making and clinical judgment in the management of this rare ectopic localization [24,35,36,37,57].

#### 3.5.6. Therapeutic Patterns in Abdominal and Ligamentous Pregnancy

In abdominal and ligamentous EPs, the literature almost universally identifies surgical management as the central therapeutic modality, given the high risk of massive hemorrhage and significant maternal morbidity [41,42,43]. One of the most frequently described therapeutic challenges relates to management of the placenta, particularly in advanced gestations. Several review articles describe a strategy of leaving the placenta in situ in situations where its removal would carry an unacceptably high risk of fatal bleeding, necessitating prolonged and careful postoperative patient follow-up [41,42,44]. However, the literature emphasizes that such approaches are based almost exclusively on individual case reports and small series, without standardized therapeutic protocols or clearly defined algorithms [41,42,43,44].

#### 3.5.7. Therapeutic Patterns in Heterotopic Pregnancy

In heterotopic pregnancy, therapeutic management most commonly involves surgical removal of the ectopic component, with efforts to preserve the intrauterine pregnancy whenever possible [43,45]. Medical treatment with systemic MTX is generally not applied in this context because of its well-known adverse effects on the intrauterine gestation [43,45]. Available sources indicate that therapeutic outcomes largely depend on early diagnosis and availability of minimally invasive surgical techniques, particularly in patients following ART use, in whom the incidence of heterotopic pregnancy is increased [21,43,45].

## 4. Discussion

### 4.1. Why Rare Ectopic Pregnancies Are Clinically and Diagnostically Different from Standard Tubal Pregnancy

Rare EPs represent a distinct clinical entity that differs fundamentally from standard tubal EP, most commonly the ampullary type, on which the majority of existing diagnostic and therapeutic algorithms have been developed [8,9,10,11]. The current literature indicates that these differences primarily arise from the specific anatomical and vascular characteristics of the implantation site, whereas gestational age and trophoblastic biological behavior (parameters frequently used as indicators of progression and risk in tubal EP) have substantially lower clinical predictive value in rare localizations [5,7,40]. A defining feature of rare EPs is that the risk of severe, potentially life-threatening complications depends predominantly on the site of implantation rather than on the size or duration of the pregnancy [5,7,40]. Implantation in regions such as a cesarean section scar, the interstitial portion of the fallopian tube, or the cervical stroma permits early and deep trophoblastic invasion into highly vascularized tissue, often in the absence of an adequate decidual barrier [7,40,50]. Literature has further expanded the concept of scar-related EP beyond classic cesarean scar implantation. A recent scoping review demonstrates that ectopic implantation may occur in a variety of uterine scar types, including scars following myomectomy and other uterine surgeries, emphasizing that abnormal implantation in scarred myometrium represents a broader clinical entity rather than an isolated phenomenon limited to cesarean delivery [51]. These findings further support the concept that implantation site–specific anatomy and local myometrial integrity, rather than epidemiological frequency alone, are the primary determinants of clinical risk, diagnostic complexity, and therapeutic decision-making in rare EPs [7,40,51]. Massive hemorrhage or uterine rupture may occur already in early gestation, challenging the traditional assumption that early pregnancy is inherently associated with a low risk of serious complications [5,7,40]. This pathophysiological and anatomical specificity explains why diagnostic and therapeutic algorithms developed for standard tubal EP cannot be directly applied to rare localizations and underscores the need for an individualized, localization-adapted clinical approach [5,7,40]. An additional diagnostic challenge arises from the fact that intrauterine implantation does not necessarily equate to a physiological pregnancy [7,40]. Entities such as CSP or cervico-isthmic pregnancy are formally located within the uterus but behave clinically as EPs due to the abnormal relationship between the gestational sac, the myometrium, and surrounding vascular structures [29,40,50]. In early stages, these implantations may be misinterpreted as low-lying intrauterine pregnancies, creating a false sense of clinical security [21,40,50]. Clinical presentation further complicates the diagnostic process. Available evidence indicates that early manifestations of rare EPs are frequently nonspecific and often indistinguishable from other forms of early pregnancy, including non-progressive intrauterine pregnancy or ongoing miscarriage [5,7,15]. In the absence of clear clinical “red flags,” accurate diagnosis relies heavily on the level of clinical suspicion and the quality of the initial TVUS assessment, which may result in delayed precise diagnosis and localization of implantation [7,15,21]. The dominant “tubal diagnostic mindset” in routine clinical practice represents an additional source of diagnostic error. Most early-pregnancy algorithms focus on the adnexa and typical sonographic signs of tubal EP, such as an adnexal mass or free fluid in the pouch of Douglas [7,15,21]. In uterine EPs, where pathological implantation occurs within or adjacent to the uterus, this approach may lead to overlooked findings, particularly if TVUS is not systematically extended to evaluate the lower uterine segment, cervix, and the region of a previous cesarean section [21,40,50]. Terminological imprecision constitutes another important contributor to diagnostic confusion, particularly with respect to implantations in the interstitial portion of the fallopian tube [29,30,32]. Inconsistent use of the terms interstitial, angular, and implantations associated with uterine cornual anomalies in both the literature and clinical practice is not merely an academic issue but has direct clinical implications, as these entities represent distinct anatomical and clinical conditions requiring different diagnostic and therapeutic considerations [29,30]. Incorrect classification may result in underestimation of rupture risk, inappropriate selection of therapeutic strategy, and suboptimal surgical planning, thereby further increasing patient risk [29,30,31]. Taken together, these considerations indicate that rare ectopic pregnancies require a higher level of clinical suspicion and an individualized diagnostic framework that extends beyond algorithms designed for standard tubal EP [5,7,40]. Recognition of these fundamental differences provides the conceptual basis for understanding the specific diagnostic pitfalls and systemic errors encountered in clinical practice, which are addressed in the subsequent section of the discussion.

### 4.2. Diagnostic Pitfalls and Systematic Errors in Clinical Practice

#### 4.2.1. Limitations in the Interpretation of β-hCG Dynamics

Interpretation of serum β-hCG dynamics represents a frequent source of diagnostic error in rare EPs. Although a suboptimal rise or plateau is traditionally associated with tubal EP, β-hCG kinetics in non-tubal locations significantly overlaps with patterns observed in tubal ectopic and early intrauterine pregnancies [7,15,16]. In cesarean scar, cervical, and interstitial pregnancies, early trophoblastic invasion into highly vascularized myometrial or cervical tissue may sustain an apparently appropriate hormonal rise de-spite pathological implantation [7,16]. Therefore, absolute β-hCG values and short-term dynamics lack sufficient discriminatory value regarding implantation sites and do not reliably predict the risk of rupture. Clinical instability in these entities depends primarily on the anatomical site of implantation and its relationship to surrounding vascular structures, rather than on laboratory parameters [15,16]. Moreover, the minimal expected rise in β-hCG over 48 h demonstrates wide biological variability and cannot be used as an absolute threshold to exclude EP. While classical algorithms are based on percentage in-creases, clinical practice shows that both viable intrauterine pregnancies and ectopic im-plantations may deviate from these limits, further reducing the specificity of this parameter [15,16]. Particularly risky is reliance on the so-called “discriminatory zone” of β-hCG as an argument against EP, as both tubal and non-tubal EPs have been documented at values below and above traditionally accepted thresholds. Therefore, β-hCG should not be used as a criterion for assessing safety from rupture nor as a basis for postponing targeted US localization in the presence of clinical–laboratory inconsistency [15,16].

#### 4.2.2. Premature Diagnostic Closure

Premature “closure” of the diagnostic process represents one of the key systemic mechanisms underlying delayed diagnosis of rare EP locations. This phenomenon occurs when an initial diagnosis is accepted as definitive before a complete and systematic anatomical evaluation has been performed. In patients following ART, visualization of an intrauterine gestational sac may lead to implicit exclusion of an ectopic component, despite a significantly increased risk of heterotopic pregnancy compared with spontaneous cycles [7,21,43]. The presence of an intrauterine pregnancy does not exclude a concurrent extra-uterine implantation. Systematic adnexal assessment must be performed independently of the intrauterine finding. Similarly, classification as PUL with stable or rising β-hCG values may result in prolonged expectant follow-up without repeating targeted evaluation of the lower uterine segment, interstitial region, or cervix [15,21]. In rare ectopic locations, where the risk of rupture depends on anatomical implantation characteristics rather than gestational age, delayed reassessment may lead to sudden hemodynamic deterioration. An additional contributing factor is reliance on initial hemodynamic stability as an argument against urgent diagnostics, although in interstitial and abdominal locations clinical de-compensation may be sudden and unpredictable. Risk reduction requires structured follow-up protocols, clearly defined criteria for repeat TVUS, and consistent anatomical verification of gestational structure localization [21,43].

#### 4.2.3. Ultrasonographic Mechanisms of Missed Tubal and Non-Tubal Ectopic Pregnancy

In tubal EP, missed diagnosis most commonly occurs in early gestation when adnexal structures are small and below the threshold of sonographic resolution or morphologically similar to corpus luteum cyst [21,40,61]. A pseudogestational sac within the uterine cavity may create a false impression of intrauterine pregnancy if specific criteria for a true gestational sac are not confirmed, including the presence of a yolk sac and the characteristic double decidual sac sign. Inadequately systematic evaluation of both adnexa and the pouch of Douglas remain a frequent technical cause of missed tubal EP [21,40]. In non-tubal locations, diagnostic challenges arise from misinterpretation of implantation within or proximity to the uterus. CSP may be misclassified as a low-implanted intrauterine pregnancy or as an ongoing miscarriage if the thickness and continuity of the myometrium between the gestational sac and the uterine serosa are not assessed [40,50]. Interstitial pregnancy may be labeled as intrauterine if continuity and adequate thickness of the surrounding myometrial layer is not analyzed, as well as its precise relationship to the endometrial cavity [29,30,31,32]. Cervical pregnancy may be interpreted as a miscarriage in progress, particularly in the presence of vaginal bleeding, if absence of the sliding sign and the presence of marked peri trophoblastic vascularization within the cervical stroma are not documented [10,11,40]. Visualization of a gestational sac within the uterus does not constitute proof of intrauterine implantation. It is essential to document continuity and adequate thickness of the myometrium surrounding the entire sac, as well as its clear relationship to the endometrial cavity. In selected cases, three-dimensional US and detailed Doppler analysis may contribute to more precise assessment of the relationship between the gestational structure, the myometrium, and the vascular pattern, particularly when interstitial or CSP is suspected. Reduction of diagnostic omissions requires a standardized and systematic US protocol that includes evaluation of the uterine cavity in at least two orthogonal planes, assessment of the lower uterine segment and postoperative scar, detailed bilateral adnexal examination, evaluation of free fluid, and documentation of the peri trophoblastic vascular pattern [21,40,50].

#### 4.2.4. Diagnostic Pitfalls in Abdominal Ectopic Pregnancy and Strategies to Avoid Misdiagnosis

Abdominal EP represents a rare but potentially life-threatening implantation site, associated with a high risk of massive intra-abdominal hemorrhage [60,61]. It may be primary (direct implantation on the peritoneum, ovary, mesentery or omentum) or secondary, following tubal rupture and abdominal reimplantation. Differentiation between primary and secondary abdominal pregnancy is clinically relevant, as vascular risk depends primarily on the site of placental implantation and its relationship to major abdominal vessels rather than on the mechanism of implantation itself [62]. The gestational structure may lie in close anatomical proximity to the uterus, creating a false impression of intrauterine localization if myometrial continuity is not analyzed [41,63]. Hemoperitoneum is often automatically attributed to ruptured tubal pregnancy without consideration of primary or secondary abdominal implantation, particularly in the absence of a clearly de-fined tubal lesion [42,59]. The key ultrasonographic criterion is documentation of the absence of a myometrial layer surrounding the gestational sac. Visualization of an embryo or gestational structure outside the anatomical boundaries of the uterus, without interposed myometrium, must raise suspicion of abdominal implantation. Additional signs include atypical placental location, unusual relationships to bowel loops or peritoneal structures, and tenderness during probe manipulation [41,60,63]. Management of placental tissue represents a particular challenge, as attempts at complete placental removal may result in catastrophic hemorrhage if the placenta is attached to major blood vessels or highly vascularized structures. Therefore, precise preoperative localization is crucial for planning the surgical strategy. TVUS may be insufficient for complete anatomical orientation. The transabdominal approach provides a broader field of assessment, while in hemodynamically stable patients with inconclusive findings, adjunct radiologic evaluation (e.g., MRI) may contribute to precise preoperative localization and surgical planning [42,60]. Due to the possibility of implantation in highly vascularized structures (omentum, mesentery, major blood vessels), early multidisciplinary assessment and planning of intervention in a facility with adequate surgical and transfusion support are recommended [62,63].

### 4.3. Therapeutic Challenges and Limitations of Standard Algorithms

Therapeutic management of EP has historically been shaped by experience derived from tubal EP, for which well-defined algorithms exist. The majority of studies consistently emphasizes that these frameworks cannot be directly applied to rare ectopic localizations, primarily due to differences in implantation anatomy, vascularization, and trophoblastic invasion patterns [5,7,40]. As a result, uncritical transposition of standard tubal algorithms to rare EPs is frequently associated with suboptimal outcomes or an increased risk of serious complications. One of the central therapeutic challenges concerns the role of MTX. Although MTX remains the cornerstone of conservative treatment in carefully selected tubal EPs, multiple sources document clear limitations of systemic MTX in rare localizations [5,7,40]. In CSP, cervical pregnancy, and interstitial pregnancy, trophoblastic tissue often demonstrates deep invasion into highly vascularized structures, even at relatively low β-hCG levels. This reduces the effectiveness of systemic MTX while simultaneously increasing the risk of delayed or massive hemorrhage [40,48,50]. Systemic MTX as monotherapy is frequently inadequate, whereas local intragestational MTX administration represents a conceptually distinct approach and is more commonly applied as part of combined treatment strategies [40,42,47]. Recent systematic evidence further confirms the limitations of systemic MTX as monotherapy in CSP. A recent systematic review demonstrates that hysteroscopic management, when applied in carefully selected and hemodynamically stable patients, is associated with favorable clinical outcomes and acceptable safety profiles. In contrast, isolated systemic MTX therapy is frequently linked to a prolonged disease course, slower resolution, and a higher likelihood of requiring additional therapeutic interventions [52]. These findings further support contemporary therapeutic strategies that favor localization-guided, procedure-based approaches in the management of CSP [52,54]. Hemostasis emerges as the primary therapeutic objective, in some cases outweighing both trophoblastic elimination and fertility preservation [40,50,52]. This principle is particularly evident in CSP and cervical pregnancy, where the risk of sudden, uncontrolled bleeding dominates the clinical course. Consequently, therapeutic planning must prioritize hemorrhage control through surgical, adjuvant, or combined approaches, as purely cytotoxic treatment strategies carry an unacceptably high risk in these settings [40,42,48,52]. Reflecting these considerations, combined and multimodal therapeutic strategies constitute the dominant pattern in literature on rare EPs. Numerous reports describe individualized approaches incorporating surgical management, local MTX administration, and, in selected cases, interventional radiological procedures [40,42,46,47,52,54]. The absence of a single “optimal” treatment should therefore not be interpreted as a lack of evidence, but rather as a consequence of the marked heterogeneity of rare EPs and the need for localization-specific decision-making based on implantation site, hemodynamic stability, and reproductive goals. Minimally invasive surgery, particularly laparoscopic techniques, is preferred therapeutic option for many rare ectopic localizations in hemodynamically stable patients [30,31,34]. While the benefits of laparoscopy in terms of reduced morbidity and potential fertility preservation are well documented, the literature emphasizes that this approach is not universally applicable. Hemodynamic instability, advanced implantation, or suspicion of massive hemorrhage remain clear indications for open surgical management [30,31,41]. Importantly, therapeutic decisions in rare EPs are closely linked to long-term reproductive implications. In implantations involving a cesarean scar and other myometrial or uterine wall localizations, treatment-related disruption of myometrial integrity may increase the risk of abnormal placentation or uterine rupture in subsequent pregnancies [29,30,40,47,52,54]. These long-term considerations further complicate therapeutic decision-making and necessitate careful balancing between immediate clinical safety and future reproductive outcomes. Finally, the formulation of robust therapeutic recommendations is limited by the quality of available evidence, which continues to derive predominantly from case reports, small series, and heterogeneous reviews [5,7,41]. Individualized therapy does not represent a departure from evidence-based medicine, but rather a rational and necessary response to the methodological constraints. The localization-dependent role and limitations of MTX in the management of rare EPs are summarized in Table 5.

### 4.4. Fertility and Long-Term Implications of Treatment

Assessment of fertility and long-term reproductive outcomes after rare EPs remains particularly challenging due to the limited and highly heterogeneous evidence base in whichfertility rarely defined as a primary outcome, necessitating cautious interpretation of long-term effects within individual clinical contexts [5,40,47,57,58,59,60]. Current evidence suggests that reproductive outcomes are determined primarily by the combination of implantation site and therapeutic approach rather than by the occurrence of EP itself [5,7]. Surgical interventions involving the uterine musculature may have a greater impact on myometrial integrity compared with more conservative or locally targeted strategies. However, robust data allowing reliable comparison or ranking of treatment modalities with respect to fertility preservation are lacking [5,41,58,59,60,61]. CSP warrants particular attention, as multiple reviews and consensus documents suggest that it may represent an early manifestation of implantation disorders associated with the placenta accreta spectrum in subsequent pregnancies [40,42,47]. Increased risks of recurrent scar implantation, abnormal placentation, and uterine rupture have been reported, although existing evidence does not allow clear differentiation between the effects of implantation localization itself and those related to the applied treatment [40,47,52,53]. Interstitial and cesarean scar implantations may carry long-term implications for uterine wall integrity. Surgical procedures such as cornuotomy or cornual resection may result in localized myometrial weakening, theoretically increasing the risk of uterine rupture in future gestations [29,30,31]. Nevertheless, evidence regarding optimal reconstruction techniques and their long-term reproductive impact remains limited and is predominantly based on small series without adequate follow-up [5,30]. In contrast, long-term reproductive outcomes following ovarian and abdominal EPs are even less well defined. Available reports indicate that fertility in these cases is more strongly influenced by underlying reproductive pathology and pre-existing risk factors than by the ectopic implantation or its treatment [35,41]. Given the extreme rarity of these entities, sufficiently robust data to support definitive conclusions are currently unavailable. In clinical practice, reproductive counseling after rare EPs should therefore be individualized, integrating available evidence, implantation site, therapeutic approach, and patient-specific reproductive goals [5,40,47]. The absence of standardized recommendations regarding optimal interpregnancy intervals and follow-up protocols further underscores the need for a cautious, personalized approach [5,47]. The lack of systematic long-term follow-up and standardized reporting of fertility outcomes represents a major limitation of the current literature, highlighting the need for structured registries and longitudinal data collection to inform future clinical guidance [7,41]. In addition to diagnostic and therapeutic challenges, the psychological impact of rare EP should not be underestimated. Loss of a desired pregnancy, often occurring after a period of uncertainty and intensive monitoring, may be associated with significant emotional distress, grief reactions, and anxiety regarding future fertility. Recognition of these psychological aspects and provision of appropriate counseling and support represent an important component of comprehensive patient care in the management of rare EPs [9].

### 4.5. Role of PUL Algorithms in the Prevention of Severe Outcomes in Rare Ectopic Pregnancies

In contemporary clinical practice, PUL represents an essential framework for the safe management of early pregnancy. Structured PUL management, combining serial serum β-hCG assessment with repeated TVUS, facilitates earlier recognition of atypical implantation patterns and non-standard growth trajectories, particularly in pregnancies that later declare as rare ectopic localizations [7,15,16]. Importantly, PUL should not be interpreted as a distinct ectopic localization, but rather as a temporary diagnostic classification that encompasses a heterogeneous group of early pregnancy outcomes, including normally developing intrauterine pregnancy, failing pregnancy, and EP [1,7,15,17]. When rare EP is suspected, standard PUL algorithms require qualitative modification rather than replacement. In this context, lower thresholds for repeat TVUS, earlier reassessment of the initial diagnostic assumption, and targeted evaluation of high-risk implantation sites—including the lower uterine segment, uterotubal junction, and prior cesarean section scar—are emphasized. Reliance on isolated β-hCG dynamics should be reduced, and greater weight should be given to anatomical findings and clinical context. Importantly, prolonged expectant follow-up that may be acceptable in typical tubal PUL should be avoided when clinical, historical, or ultrasonographic features raise suspicion of rare ectopic localization [7,15,16,21]. Although predictive models based on β-hCG dynamics may assist in risk stratification within the PUL population, their applicability in rare ectopic localizations remains limited [16,21,23]. These models do not account for anatomical implantation sites or localization-specific growth patterns and therefore cannot serve as a standalone basis for therapeutic decision-making [16,21]. The temporal dimension constitutes an additional and often underestimated component of PUL management. While short-term observation is frequently safe in typical tubal EP, prolonged diagnostic delay may carry a disproportionately high risk in rare localizations, particularly in CSP and interstitial pregnancies [7,29,40]. Accordingly, modern literature emphasizes a low threshold for repeat TVUS and early reassessment of the initial diagnostic assumption when clinical or laboratory trajectories deviate from expected patterns. PUL algorithms do not replace clinical suspicion but rather structure and enhance diagnostic safety, allowing systematic monitoring of early pregnancy without premature exclusion of high-risk ectopic entities [7,15,16].

### 4.6. Limitations of Available Evidence and Implications for Future Research

Analysis of the available literature indicates that the formulation of robust and universally applicable clinical recommendations is possible, but it is seriously constrained by the structure of the existing evidence. This pattern of evidence is not the result of methodological negligence but rather directly reflects the low incidence and pronounced clinical heterogeneity of these entities, necessitating cautious interpretation of published findings, particularly when attempting direct comparisons of diagnostic and therapeutic strategies [5,7,41]. Improved terminological consistency, uniform use of definitions, and standardized US descriptions would reduce data heterogeneity and facilitate the development of high-quality meta-analyses. These issues do not affect diagnostic interpretation alone but also extend to the reporting of therapeutic outcomes, further complicating the integration of higher-level evidence [29,30,32]. An additional limitation is that reported therapeutic outcomes in most studies are primarily focused on short-term or technical endpoints, such as initial hemorrhage control or procedural success. Long-term outcomes (including fertility preservation, recurrence risk, and abnormal placentation in subsequent pregnancies) are rarely systematically assessed or consistently reported [5,41]. Improved understanding of the true long-term impact of specific therapeutic strategies would enable more reliable ranking of treatment options with respect to reproductive outcomes. Publication bias represents another important limitation and is likely more pronounced in the field of rare EPs than in more common clinical entities. Successful or innovative therapeutic approaches are more likely to be published, whereas complications, adverse outcomes, and treatment failures are probably underrepresented, potentially leading to an overly optimistic perception of therapeutic effectiveness in the literature [5,7,41]. Differences in institutional resources, levels of expertise, and local clinical practice result in substantial heterogeneity in patient management, further limiting the generalizability of published findings across different clinical settings [5,7,40]. Although such variability does not necessarily imply inadequate care, it represents a significant obstacle to data aggregation and the performance of meaningful comparative analyses. In light of these limitations, contemporary literature increasingly emphasizes that future progress in the field of rare EPs is more likely to depend on terminological standardization and systematic data collection than on the design of randomized clinical trials, which are difficult to implement in this population [5,7,41]. Multicenter registries, uniform reporting of diagnostic criteria and therapeutic outcomes, and structured long-term follow-up are recognized as more feasible and potentially more impactful strategies for strengthening the evidence base. In the absence of high-quality comparative studies, clinical guidelines and consensus documents issued by professional societies remain a central pillar of clinical decision-making in this field. Their value lies in the systematic synthesis of available evidence combined with cumulative expert experience and regular updates reflecting emerging data—an approach of particular importance in a domain that continues to be characterized by limited and fragmented evidence [1,42,47]. An overview of key guidelines and consensus documents, together with their limitations in the context of rare ectopic localizations, is presented in Table 6.

### 4.7. Additional Conceptual and Clinical Aspects of Rare Ectopic Pregnancies in Contemporary Literature

Extremely rare forms associated with congenital Müllerian anomalies have also been described, such as pregnancy in a rudimentary uterine horn [1,55,56,64]. Although these entities account for a negligible proportion of all EPs, their clinical relevance lies in the exceptionally high risk of rupture, which most commonly occurs in the second trimester and may result in catastrophic outcomes [37,38,65]. Available reports indicate that diagnosis is frequently delayed and often established intraoperatively, despite advances in TVUS and MRI, underscoring the importance of maintaining clinical awareness of atypical implantations in patients with unusual clinical or anatomical findings [37,38,61,66,67]. In this context, European literature further emphasizes the importance of national and regional recommendations in improving diagnostic and therapeutic strategies for rare and non-tubal EPs. Published guidelines issued by the German, Austrian and Swiss societies of gynecology and obstetrics (DGGG/OEGGG/SGGG) provide a structured framework for diagnostic and therapeutic decision-making in early pregnancy, with particular emphasis on individualized management based on implantation site, hemorrhagic risk assessment and availability of multidisciplinary expertise. These recommendations highlight the limitations of standard algorithms developed for tubal EP and underscore the need for localization-oriented therapeutic strategies in rare implantations. In addition, the guidelines emphasize the importance of timely referral to centers with appropriate surgical and interventional expertise when rare or high-risk implantation sites are suspected [56]. Data derived from French national clinical practice, based on analyses of centers affiliated with the National College of French Gynecologists and Obstetricians (CNGOF), demonstrate considerable heterogeneity in the management of non-tubal EPs and emphasize the importance of multidisciplinary evaluation and individualized treatment. These findings further support the need for improved standardization of diagnostic criteria and therapeutic approaches, as well as the development of clearer guidance addressing the specific challenges of rare EP localizations [58]. Regarding long-term reproductive outcomes, recent review articles consistently indicate that most available data continue to pertain predominantly to standard tubal EP. Evidence on fertility following rare ectopic localizations remains fragmented, methodologically limited, and insufficient to clearly differentiate the effects of implantation site from those related to the applied therapeutic approach [56,62,63,64]. An additional, frequently underappreciated aspect of modern literature concerns patient counseling and interpretation of laboratory parameters in clinical decision-making. Although serum β-hCG values and their dynamics are useful tools in the initial assessment of early pregnancy, excessive reliance on laboratory thresholds may lead to erroneous clinical reasoning, particularly in atypical implantations. Accordingly, patient counseling should be based on integration of laboratory findings, TVUS, and clinical context, with explicit communication of diagnostic uncertainty and potential risks [56,58,62,63,64,65,66]. In the context of ART, some studies have examined differences in EP incidence according to IVF cycle type, including comparisons between fresh and frozen embryo transfers. While these data contribute to understanding overall ectopic risk in assisted reproduction populations, their relevance to rare ectopic localizations remains limited, as most studies do not provide detailed implantation site analysis or localization-specific clinical outcomes [25,58,60,67,68]. Finally, a substantial proportion of the literature on EP (including rare localizations) continues to be affected by systematic methodological weaknesses. Publication bias, selective reporting, and the predominance of small, uncontrolled studies may lead to overestimation of the effectiveness of diagnostic and therapeutic approaches, a limitation that is particularly pronounced in low-incidence conditions such as rare EPs [25,34,68,69,70,71].

## 5. Conclusions

Rare EPs represent a heterogeneous group of clinical conditions that, despite their low incidence, carry a disproportionately high risk of diagnostic delay, severe complications, and the need for urgent intervention. Available evidence consistently indicates that precise localization of implantation is a fundamental prerequisite for safe and rational clinical decision-making. Diagnostic and therapeutic approaches developed for tubal EP cannot be directly applied or extrapolated to rare and non-standard localizations, underscoring the need for heightened clinical suspicion, systematic and disciplined TVUS evaluation, and strict terminological consistency. Within this context, PUL plays a central role in the early recognition of rare ectopic entities when applied as an active and structured diagnostic framework rather than as a passive postponement of clinical decision-making. Management of rare EPs must be individualized and, in many cases, multidisciplinary, with control of hemorrhage and maintenance of hemodynamic stability remaining the primary therapeutic priorities, while fertility preservation represents an important goal. In the absence of high-quality comparative studies, clinical guidelines and consensus documents issued by professional societies currently provide the most reliable framework for clinical decision-making. Nevertheless, their application requires careful adaptation to individual clinical scenarios, taking into account available resources and levels of clinical expertise. Future progress in this field will depend on improved standardization of diagnostic criteria and therapeutic reporting, establishment of multicenter registries, and systematic long-term follow-up of patients with rare EPs. Such efforts are essential to strengthen the evidence base and to enable safer, more precise, and more rational management of these potentially life-threatening conditions.

Taken together, current evidence indicates that safe management of rare ectopic pregnancies depends primarily on precise localization of implantation and individualized clinical decision-making rather than extrapolation of standard tubal algorithms. Structured application of the PUL concept and systematic ultrasound evaluation remain central to early recognition and prevention of severe complications. Future progress will rely on improved standardization of diagnostic and therapeutic reporting and on the development of multicenter prospective datasets to better define optimal fertility-preserving strategies.

## Figures and Tables

**Table 1:** Standardized terminology.

Term	Anatomical Definition	Classification
Interstitial pregnancy	Implantation within the intramural portion of the fallopian tube at the uterotubal junction	Ectopic pregnancy
Cornual pregnancy	Implantation within a rudimentary or anomalous uterine horn (Müllerian anomalies)	Ectopic pregnancy
Angular pregnancy	Implantation within the endometrial cavity at the uterine angle, medial to the uterotubal junction	Intrauterine pregnancy

Note: This table summarizes standardized anatomical definitions and classification of interstitial, cornual, and angular pregnancies based on contemporary consensus recommendations and aims to reduce common terminological inconsistencies in the literature [1,3,5].

**Table 2:** Classification of Rare Ectopic Pregnancies.

Localization	Definition	Relative Frequency	Clinical Significance/Key Risks
Cesarean scar pregnancy (CSP)	Implantation of the gestational sac within the myometrial defect of a previous cesarean section	<1% of all ectopic pregnancies (increasing incidence)	Early risk of massive hemorrhage, uterine rupture, association with placenta accreta spectrum
Cervical ectopic pregnancy	Implantation within the cervical canal below the internal os	<1%	High risk of uncontrolled bleeding due to rich vascularization
Interstitial pregnancy	Implantation within the intramural portion of the fallopian tube	2–4% of all ectopic pregnancies	Late rupture with potentially massive hemorrhage
Cornual pregnancy (uterine anomalies)	Implantation in a rudimentary or anomalous uterine horn (Müllerian anomalies)	Extremely rare	High risk of rupture, often requires more radical surgical management
Ovarian pregnancy	Implantation within ovarian tissue	<1–3%	Diagnostic confusion with hemorrhagic cyst or corpus luteum
Abdominal pregnancy	Implantation within the peritoneal cavity	<1%	High maternal morbidity, “placental dilemma”
Broad-ligament/ligamentous pregnancy	Implantation between the layers of the broad ligament	Extremely rare	Difficult intraoperative diagnosis, high hemorrhagic risk
Heterotopic pregnancy	Simultaneous intrauterine and ectopic pregnancy	<1:30,000 (significantly more frequent in ART cycles)	Risk of diagnostic “closure” after confirmation of intrauterine pregnancy

Note: This table summarizes the main rare and non-tubal ectopic pregnancy localizations, their anatomical definitions, relative frequencies, and principal clinical risks based on contemporary review literature and consensus-based classifications. The term cornual pregnancy is used exclusively in the context of uterine malformations and should not be considered a synonym for interstitial pregnancy [1,5,7,8].

**Table 3:** Diagnostic Challenges in Rare Ectopic Pregnancies.

Localization	Main Ultrasound Challenges	Common Diagnostic Errors	Potential Consequences
Cesarean scar pregnancy (CSP)	Gestational sac located in the lower uterine segment, close to the scar	Misinterpretation as a low-implanted intrauterine pregnancy	Delayed treatment, massive hemorrhage, uterine rupture
Cervical ectopic pregnancy	Differentiation from ongoing miscarriage	Inappropriate curettage without hemostatic preparation	Uncontrolled, potentially fatal hemorrhage
Interstitial pregnancy	Deep intramural localization of the gestational sac	Confusion with angular or cornual pregnancy	Late rupture with massive hemorrhage
Cornual pregnancy (uterine anomalies)	Marked anatomical variability	Incorrect classification of implantation site	Inadequate surgical planning, increased risk of complications
Ovarian pregnancy	Similarity to corpus luteum cyst or hemorrhagic lesions	Failure to recognize ectopic pregnancy	Intra-abdominal hemorrhage
Abdominal pregnancy	Nonspecific or atypical ultrasound findings	Late or intraoperative diagnosis	High maternal morbidity
Heterotopic pregnancy	Presence of a confirmed intrauterine pregnancy	Exclusion of the ectopic component (“diagnostic closure”)	Rupture of the ectopic pregnancy

Note: This table summarizes the most common diagnostic challenges, frequent diagnostic errors, and potential clinical consequences associated with rare ectopic pregnancy localizations based on contemporary literature and guideline-based diagnostic frameworks [1,4,8,29,32].

**Table 4:** Therapeutic Approaches According to the Localization of Rare Ectopic Pregnancies.

Localization	Medical Therapy	Surgical Approach	Combined Approaches	Key Notes
Cesarean scar pregnancy (CSP)	Limited (local MTX only as part of combination therapy)	Transvaginal or laparoscopic resection	Local MTX + aspiration ± uterine artery embolization (UAE)	Systemic MTX as monotherapy is not recommended; high hemorrhagic risk
Cervical ectopic pregnancy	Local ± systemic MTX (selectively)	Ultrasound-guided aspiration	MTX + UAE or other hemostatic procedures	Hemorrhage control represents the central therapeutic goal
Interstitial pregnancy	Selected cases	Laparoscopic cornuotomy or resection	Surgery + local MTX	Terminological and diagnostic precision is crucial for therapy selection
Cornual pregnancy (uterine anomalies)	Limited	Resection of the rudimentary or anomalous horn	Rare	High risk of rupture, often requires more radical surgical management
Ovarian pregnancy	Rare	Wedge resection of the ovary or oophorectomy	Rare	Diagnosis is often established intraoperatively
Abdominal pregnancy	No	Surgical management	Placenta left in situ in selected cases	Requires prolonged and careful postoperative follow-up
Heterotopic pregnancy	No (for the ectopic component)	Surgical removal of the ectopic component	–	The goal is preservation of the intrauterine pregnancy when possible

Note: Therapeutic strategies summarized in this table reflect contemporary clinical practice derived from guideline-based recommendations, narrative reviews, and observational studies. Owing to the rarity and heterogeneity of non-tubal ectopic pregnancies, most recommendations are based on expert consensus and lower-level evidence rather than randomized comparative trials [1,5,7,15,40,42].

**Table 5:** Role of Methotrexate in Rare Ectopic Pregnancies (Conceptual Overview).

Localization	Systemic MTX	Local (Intragestational) MTX	Limitations/Key Notes
Cesarean scar pregnancy (CSP)	No as monotherapy	Yes	Risk of delayed or massive hemorrhage; used exclusively as part of a combined approach
Cervical ectopic pregnancy	Yes (selectively)	Yes	Prior or concomitant hemostatic preparation is required
Interstitial pregnancy	Selectively	Yes	Limited efficacy in cases of deep trophoblastic invasion; risk of rupture persists
Cornual pregnancy (uterine anomalies)	Limited	Limited	Poor therapeutic effect; surgical intervention often required
Ovarian pregnancy	Rare	Rare	Insufficient data and heterogeneous outcomes
Abdominal pregnancy	No	No	Unacceptably high risk of complications
Heterotopic pregnancy	Contraindicated	–	Risk to the intrauterine pregnancy

Note: The role of methotrexate summarized in this table is based on available observational data, narrative reviews, and expert consensus. Due to the rarity and clinical heterogeneity of non-tubal ectopic pregnancies, high-quality prospective or randomized comparative evidence remains limited, and therapeutic decisions are primarily guided by implantation site and clinical context [5,7,29,30,40,42,47,52,54].

**Table 6:** Key Guidelines and Consensus Documents Relevant to Rare Ectopic Pregnancies.

Organization	Year	Focus	Key Message	Applicability to Rare Localizations
ACOG	2018	Tubal ectopic pregnancy	Standard diagnostic and therapeutic algorithms	Limited (primarily tubal ectopic pregnancy)
RCOG	2016	All ectopic pregnancies	Differentiated approach with clinical assessment	Partial (rare localizations insufficiently addressed)
NICE	2019/2025	Ectopic pregnancy and PUL	Structured follow-up and PUL-based diagnostic algorithms	Partial (useful for PUL framework, not localization-specific)
SMFM	2022	Cesarean scar pregnancy (CSP)	Avoid expectant management and systemic MTX monotherapy; emphasize targeted treatment	High (CSP-specific recommendations)
ISUOG/VI-SUOG	2020–2024	Ultrasound diagnostics	Standardization of ultrasound criteria and structured reporting	High (crucial for early and precise localization)
ESHRE	2020	Terminology and definitions	Harmonization of nomenclature and classification of ectopic pregnancy	High (terminological and conceptual foundation)
JOGC	2021	PUL and non-tubal ectopic pregnancy	Algorithmic approach and safe follow-up strategies	Partial (useful for PUL and non-tubal framework)
German Society for Gynecology and Obstetrics (DGGG)	2023/2024	Ectopic pregnancy and early pregnancy complications	Structured diagnostic and therapeutic recommendations emphasizing individualized management and careful selection of medical and surgical treatment	High (includes non-tubal and scar-related implantations; clinically applicable)
French national practice data (CNGOF-related)	2022	Management of non-tubal ectopic pregnancies	Demonstrates heterogeneity of clinical practice and highlights need for standardized and multidisciplinary management	High (direct relevance to CSP, interstitial, ovarian and other non-tubal localizations)

Note: This table summarizes key international guidelines, consensus statements, and national recommendations relevant to the diagnosis and management of ectopic pregnancy. Although most guidelines primarily address tubal ectopic pregnancy or PUL, several recent documents increasingly recognize the clinical complexity of non-tubal and rare ectopic localizations and emphasize individualized, localization-adapted management strategies [1,8,9,10,11,42,55,56,58].

## Data Availability

Data presented in this study are available from the corresponding author upon request.

## Abbreviations

The following abbreviations are used in this manuscript:

EP	ectopic pregnancy
PUL	pregnancy of unknown location
TVUS	transvaginal ultrasound
β-hCG	beta-human chorionic gonadotropin
CSP	cesarean scar pregnancy
ART	assisted reproductive technologies
MTX	Methotrexate
US	Ultrasound
UAE	uterine artery embolization
MRI	magnetic resonance imaging
ACOG	American College of Obstetricians and Gynecologists
RCOG	Royal College of Obstetricians and Gynaecologists
NICE	National Institute for Health and Care Excellence
SMFM	Society for Maternal-Fetal Medicine
ISUOG	International Society of Ultrasound in Obstetrics and Gynecology
VI-SUOG	Vaginal and Intrauterine Ultrasound Group
ESHRE	European Society of Human Reproduction and Embryology
JOGC	Journal of Obstetrics and Gynaecology Canada
DGGG	German Society for Gynecology and Obstetrics
CNGOF	National College of French Gynecologists and Obstetricians
